# Potential of bacterial outer membrane vesicles in tumor vaccine: characteristics, advancements, and future directions

**DOI:** 10.1042/EBC20253004

**Published:** 2025-03-28

**Authors:** Yizhe Yang, Yumin Wu

**Affiliations:** 1 College of Nano Science & Technology (CNST), Soochow University, Suzhou, Jiangsu 215123, China; 2 Institute of Functional Nano & Soft Materials (FUNSOM), Soochow University, Suzhou, Jiangsu 215123, China

**Keywords:** bacterial outer membrane vesicles (OMVs), cancer immunotherapy, immunogenicity, genetic and chemical modification, tumor vaccine

## Abstract

Bacterial outer membrane vesicles (OMVs), naturally released by Gram-negative bacteria, are a type of lipid bilayer nanoparticles containing many components found within the parent bacterium. Despite OMVs were first considered mere by-products of bacterial growth, recent studies have shown them as a highly adaptable platform for tumor vaccine. Here, we first demonstrate the biogenesis of OMVs, then review the strong immunogenicity of OMVs as an immune adjuvant in tumor vaccine and its excellent vaccine delivery capability, and finally discuss OMVs’ engineering potentials through summarizing recent scientific advancements in genetic engineering, chemical modification, and nanotechnology. We also point out the clinical trials and future challenges of OMV-based vaccine. Overall, this review offers valuable insights into cancer immunotherapy, providing a roadmap for leveraging OMVs as a versatile platform for next-generation cancer vaccines.

## Introduction

Cancer remains one of the leading causes of death worldwide, accounting for nearly 10 million deaths annually [1]. Current treatments include surgery, chemotherapy, radiotherapy, and immunotherapy, which have improved survival rates in many cancer types [1,2]. However, these therapies often come with significant side effects and are less effective in advanced or metastatic cancers [3]. Additionally, issues such as drug resistance and the immunosuppressive tumor microenvironment (TME) limit the efficacy of existing treatments [4].

Tumor vaccines are a form of immunotherapy designed to stimulate the immune system to recognize and destroy cancer cells [5]. They are broadly categorized into preventive and therapeutic vaccines. Preventive vaccines aim to protect against virus-induced cancers, such as those caused byhuman papillomavirus (HPV) [6]. Therapeutic vaccines, on the other hand, target existing tumors by enhancing the immune response against tumor-associated antigens [6]. Despite significant advancements, current tumor vaccines still face challenges, including low immunogenicity, complex production processes, high development costs, and the immunosuppression of TME [7,8]. These issues underscore the need for novel vaccine carriers.

**Figure 1:**
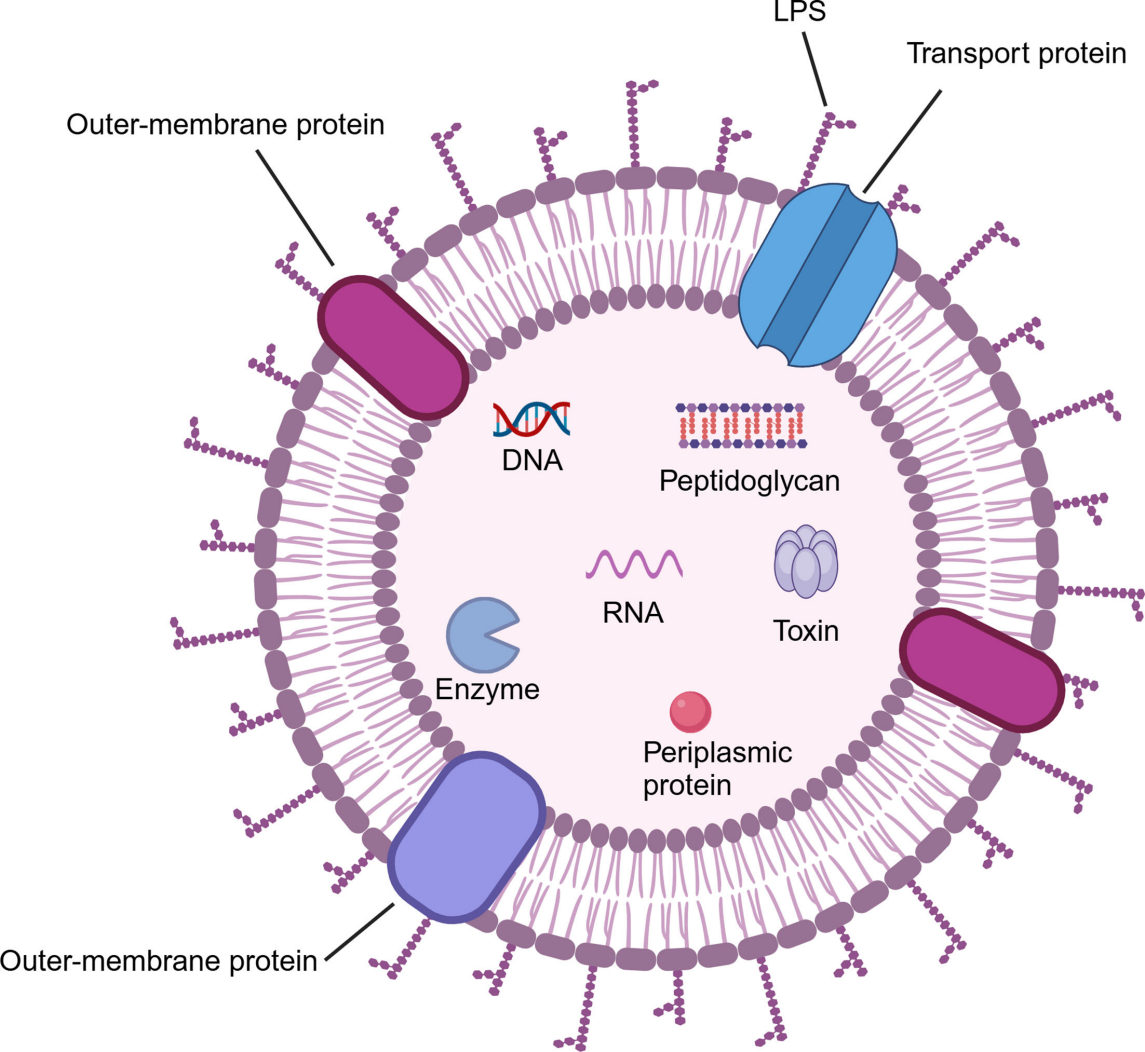
Structure and content of outer membrane vesicles. Outer membrane vesicles (OMVs) are nanoscale spherical structures (20–250 nm) secreted by Gram-negative bacteria. They encapsulate periplasmic proteins and carry membrane-bound proteins, playing crucial roles in bacterial interaction with their environment. OMVs are rich in diverse components, including lipopolysaccharides (LPS), membrane-bound proteins, enzymes (like autolysins), toxins, DNA, RNA, and peptidoglycan.

Outer membrane vesicles (OMVs), a type of lipid bilayer nanoparticles, are naturally released by Gram-negative bacteria and originate from the outer membrane (OM) [9,10]. The small spherical structures of OMVs, ranging from 20 to 250 nm in diameter, enclose periplasmic soluble proteins and bear external proteinaceous materials, which are important in bacteria–environmental interactions [11]. Studies have shown that OMVs contain lipopolysaccharide (LPS) [12], periplasmic and membrane-bound proteins [13], enzymes (such as autolysins), toxins [14], DNA [15,16,17], RNA [17], and peptidoglycan [16][18] (Figure 1).

Initially, William Coley injected killed bacteria directly into tumors and observed tumor regression, thus developing the first bacterial-based cancer therapy [19]. Following this, the concept of using bacteria as biological tumor vaccine carriers gradually gained recognition, providing a theoretical foundation for developing OMVs as cancer vaccines derived from bacterial OM [20]. At first, OMVs were thought to be merely artifacts of growth or by-products of cell lysis; however, their presence in cerebrospinal fluid from acute meningitis patients indicated that OMVs are produced outside laboratory conditions [21]. As research progressed, scientists found that OMVs stimulate both humoral- and cell-mediated immunity in a manner similar to bacteria [22,23]. However, unlike live bacteria, OMVs offer significant advantages in terms of safety and ease of production, thus paving the way for the development of genetically engineered OMV-based tumor vaccines [20]. Hence, this review aims to provide a comprehensive overview of OMV tumor vaccine, focusing on its unique immune stimulation properties, excellent capability of vaccine delivery, and engineering potentials by summarizing current advancements and key studies in this area.

## The biogenesis of OMVs

**Figure 2:**
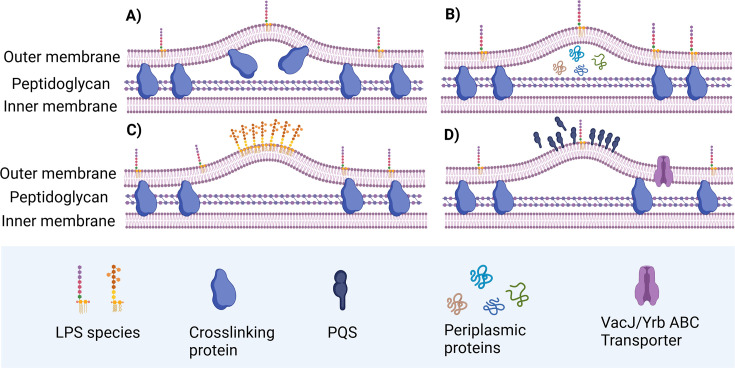
Current models of outer membrane vesicles biogenesis mechanism. **(A**) A reduction in the cross-links between the outer membrane (OM) and peptidoglycan, weakening the structural integrity of the membrane; (**B**) accumulation of periplasmic content, leading to an increased pressure that drives vesicle formation; (**C**) lipopolysaccharide (LPS) remodeling, which alters the OM’s composition and may facilitate vesicle budding; and (**D**) the bilayer-couple effect, where the insertion of biomolecules into the outer leaflet of the membrane induces curvature with the help of *Pseudomonas* quinolone signal (PQS) or VacJ/Yrb ATP-binding cassette (ABC) transporter, ultimately promoting the formation of vesicles.

**Figure 3:**
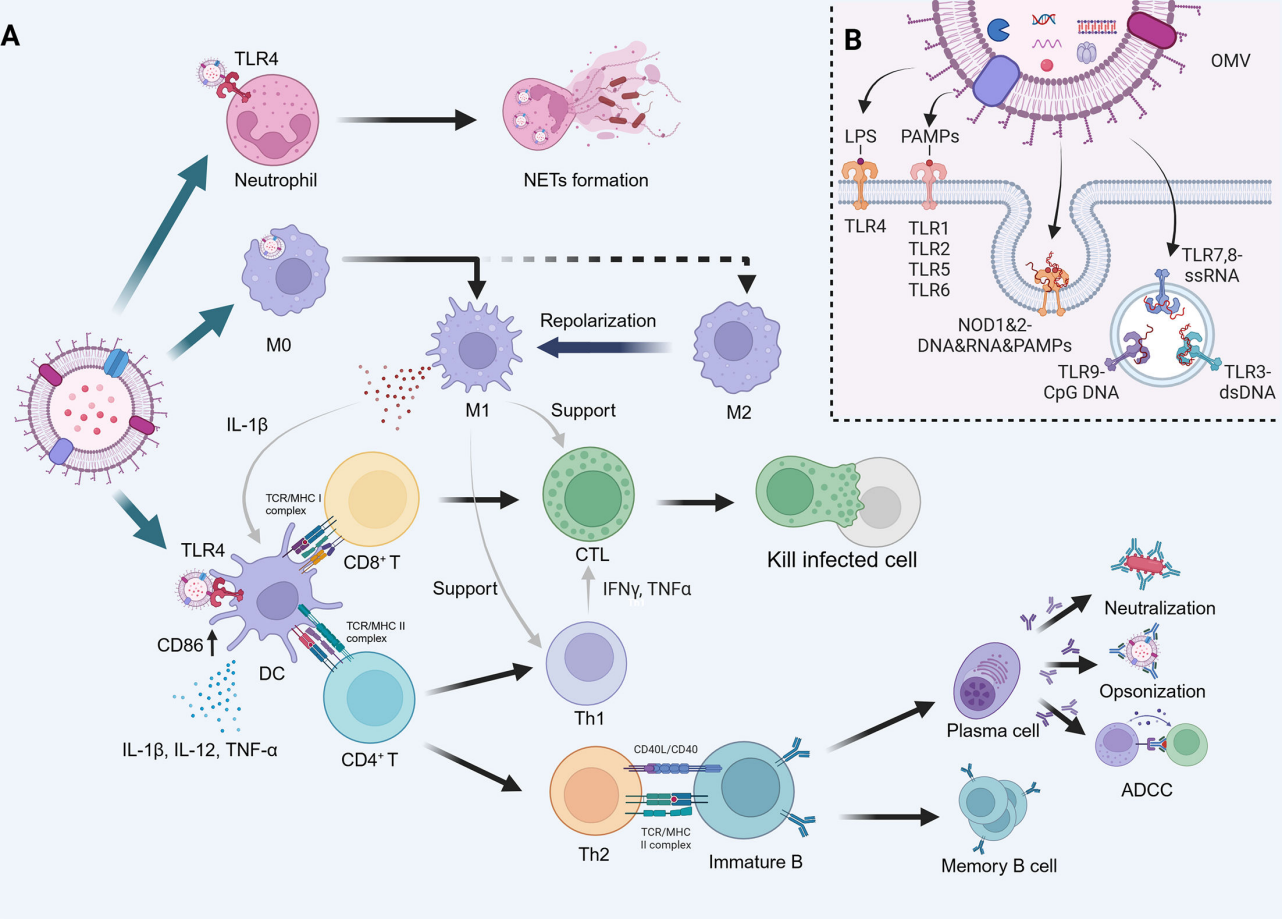
OMV-induced immune response. **(A**) Outer membrane vesicles (OMVs) can activate neutrophils via toll-like receptor 4 (TLR4), inducing the formation of neutrophil extracellular traps (NETs). They also polarize macrophages in the tumor microenvironment (TME) from the M2 to M1 phenotype, enhancing Th1- and cytotoxic T lymphocyte (CTL)-mediated cytotoxicity while secreting IL-1β to promote dendritic cell (DC) antigen presentation. Furthermore, OMVs can activate DCs through TLR4, up-regulating CD86 and secreting interleukin (IL)-1β, IL-12, and TNF-α, facilitating antigen presentation to CD8^+^ T cells, which differentiate into CTLs. Additionally, DCs present antigens to CD4^+^ T cells, driving Th1 differentiation to support CTL functions via cytokines such as IFN-γ and TNF-α, or Th2 differentiation to mediate B cell responses, including neutralization, opsonization, and antibody-dependent cellular cytotoxicity (ADCC). (**B**) OMVs’ pathogen-associated molecular patterns (PAMPs), such as lipopolysaccharide (LPS), DNA, and RNA, effectively engage pattern recognition receptors on cells. LPS specifically activates TLR4, while other PAMPs stimulate additional TLRs and NOD1 and 2 pathways, amplifying the immune response.

**Figure 4:**
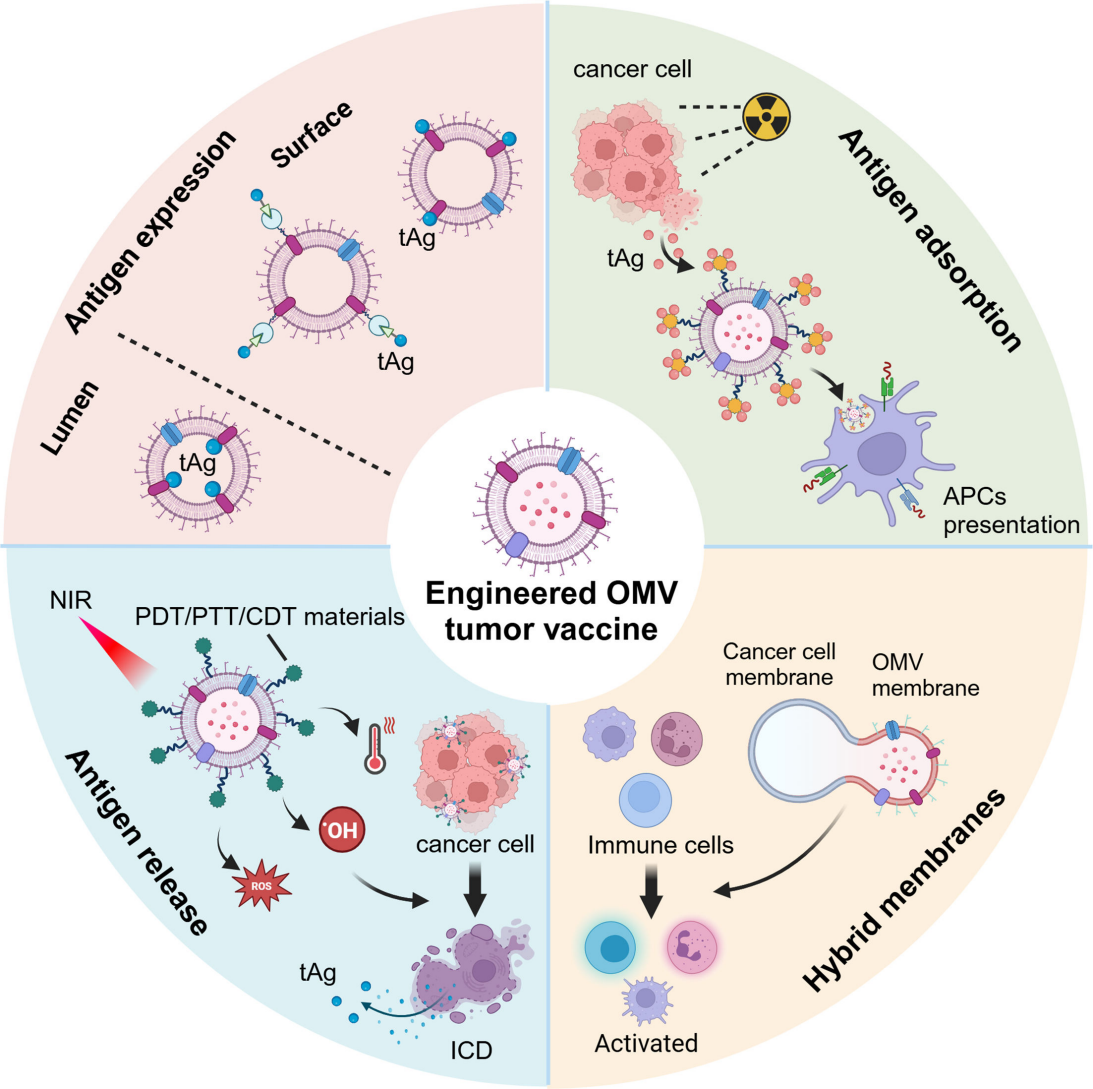
Engineered outer membrane vesicle (OMV) tumor vaccine strategies. Outer membrane vesicle (OMVs) can be tailored to display tumor antigens on their surface or encapsulate them within their lumen. They can also synergize with photodynamic therapy (PDT), photothermal therapy (PTT), or chemodynamic therapy (CDT), carrying therapeutic materials to induce immunogenic cell death (ICD) and release tumor antigens. Moreover, OMVs can be engineered to capture and present tumor antigens to APCs following radiotherapy. Finally, hybrid membranes combining OMVs and tumor cell membranes can be developed to elicit enhanced antitumor immune responses.

The biogenesis of OMVs is hypothesized to occur through a budding process from the bacterial surface, as evidenced by electron microscopy, which has observed OMVs in association with bacterial membranes [9]. Additionally, an essential characteristic of OMVs is the enrichment of specific protein and lipid cargo, such as virulence factors [24], glycoside hydrolases [25], acidic hydrolases, and alkaline phosphatases [26], which facilitates bacterial invasion and survival. However, the exact process of OMV production from bacterial membranes and its cargo selection mechanism has not been fully elucidated. Current models suggest that OMV biogenesis may result from (1) a reduction in outer membrane-peptidoglycan cross-links (Figure 2A), (2) periplasmic content accumulation (Figure 2B), (3) LPS remodeling (Figure 2C), or (4) the bilayer-couple effect, wherein biomolecule insertion into the outer leaflet of the membrane induces curvature and vesicle formation (Figure 2D) [27]. In addition, OMV cargo selection necessitates extensive compartmentalization of the OM to form specific regions from which OMVs bud. In *Porphyromonas gingivalis*, intact LPS is essential for proper cargo selection [28]. This indicates that protein–lipid interactions, possibly via direct recognition or unknown factors, guide cargo into OMVs, similar to galectin-mediated sorting in exosomes [29]. Furthermore, in *Bacteroides*, the lipoprotein export signal domain in lipoproteins is crucial for proper OMV packaging, with mutations disrupting this process, indicating its role as a key protein-sorting signal in OMVs [30].

## OMV as a tumor vaccine platform

The core of cancer vaccines lies in delivering tumor antigens along with immune adjuvants to lymphoid tissues and antigen-presenting cells (APCs), such as macrophages and dendritic cells (DCs), thereby activating the immune system to eliminate tumor cells [31,32]. In this context, OMV vaccines offer distinct advantages in cancer immunotherapy, particularly in stimulating the immune system to enhance tumor-killing capabilities. First, OMVs exhibit strong immunogenicity, which represents their most crucial characteristic comparing with other vaccine carriers such as liposomes and exosomes. Secondly, OMVs serve as an excellent platform for vaccine delivery. Finally, OMVs can be genetically or chemically modified to enhance their functionality, enabling OMVs to be integrated with other therapeutic approaches in combination treatments. Consequently, the following discussion will focus on these three characteristics of OMV-based vaccine.

### Natural immunogenicity as an immune adjuvant

First, OMVs exhibit strong immunogenicity. They primarily activate the immune system through two mechanisms: innate immune response activation and adaptive immune response initiation. Specifically, the pathogen-associated molecular patterns (PAMPs) present on OMVs, such as LPS and flagellin, can be recognized by pattern recognition receptors on immune cells (Figure 3B). This recognition process triggers the innate immune response, leading to the production of cytokines, inflammation, and programmed cell death [8] (Figure 3A).

The early stages of tumor development are typically characterized by an inflammatory TME, which attracts a large influx of immune cells that stay within the tumor tissue through a phenomenon known as chemotaxis [33]. Among these immune cells, neutrophils and tumor-associated macrophages (TAMs) are the most abundant white blood cells infiltrating various types of tumors [34,35]. M1-like TAMs, also known as classically activated macrophages, exhibit anticancer properties by releasing nitric oxide and stimulating naive T cells to generate Th1/cytotoxic responses [36]. In contrast, the tumor environment predominantly harbors M2-like TAMs or alternatively activated macrophages, which actively promote tumor proliferation, angiogenesis, metastasis, and immune evasion [36]. Notably, OMVs can reprogram macrophages by repolarizing M2-like TAMs into M1 phenotypes while stimulating them to release cytokines and chemokines such as interleukin-1β (IL-1β) and interleukin-18 (IL-18), which further modulate CD4^+^ and CD8^+^ T cell functions [37–39]. Additionally, due to the characteristics of PAMPs, OMVs can be recognized by neutrophils through toll-like receptor 4 (TLR4), mediating their delivery [40]. On one hand, neutrophils can respond to chemokines and cytokines, such as the pro-inflammatory IL-6 released by macrophages, which attract and recruit neutrophils to the infection site [41]. On the other hand, during acute infections, neutrophils play a key role in effectively phagocytosing and eliminating the pathogen [41] (Figure 3A).

Apart from innate immune response activation, adaptive immune response can be initiated by OMVs. The critical step in this process is antigen presentation by APCs, particularly through cross-presentation by DCs. Specifically, OMVs can promote DC maturation, enabling these cells to cross-present antigens and secrete cytokines that activate effector T cells, thus initiating a systemic immune response [4,42]. For example, DCs usually remain immature and fail to recognize inflammatory signals in TME, resulting in immune suppression. Study has shown that OMV administration allows surface PAMPs to interact with TLR4 on immature DCs, driving their maturation [43]. Antigen cross-presentation of DCs primarily follows two pathways: the phagosomal route [44], where antigens are degraded in phagosomes and loaded onto MHC-I molecules, and the endosomal-to-cytosolic pathway [45], where antigens are translocated to the cytosol, degraded by proteasomes, and transported to the endoplasmic reticulum for MHC-I loading. These processed antigens then activate T cells, leading to immune responses from cytotoxic and helper T cells [46]. Cytotoxic T cells can induce apoptosis in target cells via perforin and granzyme B, or caspase activation by Fas-Fas ligand interactions, while helper T cells, through CD40L interactions, stimulate B cell differentiation and antibody production, supporting neutralizing, opsonization or antibody-dependent cell-mediated cytotoxicity [38,47,48] (Figure 3A).

Nie et al. demonstrate that pre-vaccination using bacteria-derived OMVs rich in PAMPs can significantly enhance the efficacy of tumor vaccines through trained immunity. When these OMVs are administered intraperitoneally to mice, they activate inflammasome signaling pathways, triggering the secretion of IL-1β. This increased IL-1β then promotes the production of APC progenitors, thereby enhancing the immune response to tumor antigens and boosting tumor-antigen-specific T cell activation [22]. However, this strong immunogenicity should be handled carefully. Otherwise, it may lead to safety concerns. Therefore, Kim et al. studied the therapeutic effect of genetically modified OMVs and demonstrated that genetically engineered OMVs from an attenuated *Escherichia coli* strain (*E. coli msbB^−/−^, ∆msbB*) effectively suppress CT26 murine colon adenocarcinoma. By removing lipid A through *msbB* gene knockout, these OMVs can avoid TLR4 activation and associated endotoxin risks, while also showing improved yields over wildtype OMVs. They found that the modified OMVs specifically target tumor tissue *in vivo*, triggering interferon-γ (IFN-γ) and C-X-C motif chemokine ligand 10 (CXCL10) to stimulate a strong, sustained immune response that clears tumors. Notably, this antitumor effect depends on IFN-γ, as IFN-γ-deficient subjects did not show significant immune responses, and treatment showed minimal side effects, highlighting OMVs as a promising cancer therapy [23].

### Vaccine delivery capacity

OMVs exhibit excellent delivery capabilities, which are crucial for the precise targeting of tumor vaccines. First, the bilayer lipid membrane structure of OMVs allows hydrophobic drugs to bind to the lipophilic leaflets, enabling OMVs to carry various bioactive molecules like doxorubicin (DOX) [49]. Secondly, the nanoscale size of OMVs allows them to be passively targeted, through tumor-enhanced permeability and retention effect or direct drainage to lymph nodes, where they can be taken up by APCs [50]. Alternatively, OMVs can be actively targeted by expressing ligands like HER2-specific affibody via genetic engineering for precise delivery [51]. Additionally, studies have shown that OMVs possess tissue penetration capabilities. Ning et al. reported a novel OMV-based drug delivery system, I-P-OMV, with excellent stratum corneum penetration and specific targeting of melanoma [52]. Han et al. have indicated that OMVs derived from *E. coli* K1 have the potential for brain-targeted drug delivery across the blood–brain barrier (BBB). Furthermore, due to their lipid bilayer membrane, OMVs can enter host cells through: clathrin, caveolin and lipid raft-mediated endocytosis, micropinocytosis, and membrane fusion, endowing them with robust intracellular delivery capabilities [38]. Rong et al. developed a ‘hitchhiking’ strategy, where OMVs carrying DOX are selectively recognized and phagocytosed by neutrophils capable of overcoming BBB and migrating to bacteria-colonizing tumor, facilitating targeted drug delivery to gliomas [40]. Finally, OMVs can also achieve *in situ* release via bacteria. Nie and Zhao’s group developed an engineered *E. coil* that, upon oral administration, survived the gastrointestinal tract and released OMVs loading tumor antigens upon arabinose induction. These modified OMVs then penetrated intestinal barriers and triggered a strong antitumor immune response and memory in preclinical models [53].

### Engineering potential and modifiability

Empirical studies have demonstrated that vaccines exhibit enhanced efficacy when adjuvants and antigens are integrated into specific formulations, although the underlying mechanisms remain to be fully understood [54]. Leveraging the immunostimulatory properties of OMVs alongside the targeted delivery of tumor antigens, OMVs can be engineered for potential applications in tumor vaccines, where the way of loading tumor antigens (tAg) plays a key role. The main strategies for tAg loading in OMV-based tumor vaccines include (1) direct expression of tAg on OMVs, (2) induction of *in situ* tAg release, (3) *in vivo* adsorption of tAg by OMVs, and (4) tumor–OMV hybrid membranes (Figure 4). These loading methods primarily rely on genetic engineering, chemical modification, and nanotechnology.

#### Direct expression of tAg on OMVs

OMVs can be genetically engineered to express heterologous proteins tumor antigen protein, which are mainly divided into two types: surface-exposed expression and luminal expression within the OMV compartments [55]. When expressing antigens, research has shown that surface-exposed antigens may facilitate antigen-specific B cell binding [56]. In contrast, antigens located within the vesicle lumen may be shielded from B cells and are more likely to induce cytotoxic T cell responses [56]. Therefore, the desired immune response determines the design and localization of heterologous proteins within OMVs [55].

The localization strategies rely on OMVs’ unique production methods and specific protein sorting mechanisms. For surface-exposed expression, the typical approach is to fuse the cargo with proteins that are abundantly present on the OMV membrane, such as hemoglobin protease (Hbp) and cytolysin A (ClyA). Structurally, Hbp consists of a C-terminal helical domain for OM anchoring, an N-terminal signal peptide for transmembrane transport, and a central messenger domain, which can be replaced by antigen peptides [57,58]. However, this approach of antigen display appears to be limited to small protein fragments [59]. Hence, this brings to larger protein display frame like the 34-kDa pore-forming toxin ClyA [60–62]. Recent studies demonstrate that genetic fusion of recombinant proteins such as β-lactamase and green fluorescent protein (GFP) to the C-terminus of ClyA facilitates functional display of these proteins on the surface of *E. coli* and its OMVs, with its C-terminal fusion extending outward, effectively eliminating ClyA’s hemolytic oligomeric structure and showing no cytotoxicity associated with the fusion [60].

To achieve luminal expression of antigens, one approach is to localize the target protein within the periplasmic space [55]. For instance, Kesty and Kuehn managed to encapsulate GFP inside OMVs through fusing GFP gene with a twin-arginine translocation (Tat) signal sequence, enabling its transport into the periplasmic space via the Tat system in *E. coli* [63]. Alternatively, another strategy involves fusing the antigen with abundant OM proteins, such as OmpA in enteric Gram-negative bacteria, on the periplasmic side to facilitate its incorporation into the OMV lumen [64]. OmpA possesses a dual-domain structure, with the N-terminal 171 amino acids forming a β-barrel that ensures membrane insertion and stability, while the C-terminal region is localized in the periplasm [64]. A study used mutational inactivation of the MsbB (LpxM) lipid A acyltransferase to produce low-endotoxicity OMVs from *E. coli* O157 and chromosomal tagged a FLAG epitope fused to the C-terminal of truncated OmpA, enabling successful localization of FLAG within OMV lumen [65].

However, due to tumor heterogeneity, genetic and phenotypic variations among tumor cells lead to significant differences in tumor antigens between patients. This variation necessitates a flexible OMV vaccine platform that can swiftly and concurrently present multiple antigens [66]. ‘Plug-and-display’ technology is a modular platform that enables rapid and specific presentation of antigens on cell surfaces, through a peptide tag (‘tag’) binding to its protein partner (‘catcher’) by the rapid formation of an isopeptide bond between them [67,68]. Currently, two commonly used ‘plug-and-display’ systems are the SpyTag/SpyCatcher pair and the SnoopTag/SnoopCatcher pair, which are bioorthogonal [68]. Based on this technology, Nie and Zhao’s group developed a multifunctional OMV vaccine platform known as CC-OMVs by employing the two aforementioned ‘plug-and-display’ systems by fusing ‘catchers’ with ClyA on the surface of OMVs and linking various tumor antigens with different ‘Tags’. The result showed that CC-OMVs can effectively eliminated pulmonary melanoma metastases and inhibited subcutaneous colorectal tumor growth [66]. Furthermore, Nie and Zhao’s group expanded the ‘plug-and-display’ strategy to OMV-based tumor mRNA vaccine platform called ‘OMV-LL-mRNA’ [69]. Specifically, they fused the archaeal RNA-binding protein L7Ae to the C-terminus of the OMV surface protein ClyA and added a corresponding C/D binding sequence to the 3´ untranslated region of *in vitro* transcribed mRNA, which facilitated rapid mRNA adsorption onto the OMV surface [69,70]. Additionally, to enhance endosomal escape and subsequent mRNA translation, listeriolysin O (LLO) was also fused to ClyA, aiming at effective presentation of mRNA antigens to DCs for efficient processing [69,71]. Ultimately, this engineered OMV mRNA vaccine significantly inhibited melanoma and colorectal cancer progression and induced long-term immune memory [69].

#### Induction of *in situ* tAg release

Another method of antigen loading involves the induction of antigen release to create an *in situ* tumor vaccine. This approach primarily employs OMVs loaded with tumor necrosis-inducing agents or materials for photothermal therapy (PTT), photodynamic therapy (PDT), or chemodynamic therapy (CDT) to induce immunogenic cell death (ICD). This process generates damage-associated molecular patterns (DAMPs) and PAMPs, thereby converting ‘cold tumors’ into ‘hot tumors’ and effectively stimulating immune responses [8]. This method can be combined with *in vivo* antigen capture and enrichment strategies to enhance therapeutic outcomes. For example, Chen and Yang’s team developed an OMV-coated nanoplatform, HMSeN-ANX5@HOMV, where annexin A5 can be released in TME and block phosphatidylserine exposure on apoptotic cells and prevent macrophage phagocytosis, leading to secondary necrosis, tumor antigen release, and immune activation [72]. Additionally, Ning et al. engineered *E. coli* OMVs modified with an αvβ3 integrin-targeting peptide and indocyanine green for transdermal photothermal tumor necrosis factor-related apoptosis-inducing ligand (TRAIL) therapy in melanoma [52]. Additionally, Xiang et al. enhanced the tumor-targeting ability of OMVs via macrophage-mediated delivery and co-loaded photosensitizer Ce6 and chemotherapeutic drug DOX to boost antitumor efficacy [73]. This synergistic therapy showed an eradication of triple-negative breast tumors in mice without side effects and prevention of lung metastasis [73]. Furthermore, Qu et al. loaded a Fe(III)-based metal-organic framework containing metronidazole (MTD) into OMVs from *Fusobacterium nucleatum* [74]. The release of MTD and Fe *in situ* induced ICD through Fenton-like reactions and antibacterial effects, producing DAMPs and PAMPs, thereby reversing TME and activating immune responses [74].

#### *In vivo* adsorption of tAg by OMVs

After radiation or PTT, tumor cells release novel antigens, but the immune system has often limited ability to recognize these antigens effectively [75]. Therefore, capturing tumor antigens offers a promising approach for OMV-based *in situ* tumor vaccination. For example, Li et al. engineered OMVs conjugated with maleimide and loaded with 1-methyltryptophan (1-MT), called 1-MT@OMV-Mal [76]. After PTT, intratumoral injection of 1-MT@OMV-Mal enables stable thioether bonding with tumor antigens, facilitating effective DC recognition and uptake. Moreover, 1-MT@OMV-Mal overcomes indoleamine 2,3-dioxygenase-mediated immunosuppression on tumor-infiltrating T cells, resulting in marked inhibition of both primary and distant tumors [76].

#### Tumor–OMV hybrid membranes

Tumor heterogeneity presents a significant challenge in antitumor research. In the context of personalized cancer therapy, tumor cell membranes offer a promising approach due to their capacity for homologous tumor targeting and their rich content of tumor antigens [77]. Hence, it is promising to create hybrid membranes by fusing tumor cell membranes with highly immunostimulatory OMV membranes to overcome the difficulty of identifying and isolating novel tumor antigens while keeping immune stimulation in tumor vaccine [78]. In 2020, Tang et al*.* first introduced the eukaryotic–prokaryotic vesicle hybrid strategy, where melanoma cell membrane vesicles were fused with OMVs from attenuated *Salmonella*, serving as a prophylactic vaccine to stimulate the immune system and trigger an antitumor response [79]. Similarly, Zhang et al*.* developed a novel functional vesicle (mTOMV) by fusing bacterial OMVs with tumor-derived cell membranes (mT), which improved the specific lytic capability of T cells against homologous tumors and effectively inhibited lung metastasis [80].

## Clinical trials and future challenges

Currently, very few OMV-based vaccines have entered clinical trials, with most remaining at the research and animal testing stages. The emerging OMV tumor vaccines are largely still in the laboratory validation phase. The first and only marketed OMV-based vaccine is Bexsero, which demonstrates broad protection against various *Neisseria meningitidis* serogroup B strains [81]. The US Food and Drug Administration has also granted GlaxoSmithKline approval for a Phase II clinical trial of their experimental *N. gonorrhoeae* vaccine (NgG), which uses naturally blebbed small polysaccharide-protein OMVs known as generalized modules for membrane antigens [82]. While other OMV-based vaccines for infections of Gram-negative bacteria such as *Shigella flexneri* and *Haemophilus influenzae* are under development, none have yet entered clinical trials [83,84]. Additionally, clinical studies on intranasal OMV vaccines, conducted by the Walter Reed Army Institute of Research and the Norwegian Institute of Public Health, demonstrated increases in serum and nasal antibody titers after intranasal vaccination comparable to intramuscular injection, with good tolerance and no nasal inflammation [85,86]. Despite numerous advantages, OMV-based tumor vaccines still face several challenges in future application.

### High reactogenicity of PAMPs on OMVs

OMVs naturally released from bacteria often contain high levels of endotoxin LPS, which can trigger excessive immune responses [87]. One solution is to use detergents (e.g., deoxycholate) to chemically extract OMVs from the whole bacteria, which increases OMV yield and significantly reduces LPS and lipoprotein content, thereby lowering immunogenicity and enhancing OMV tolerance [88–90]. However, this can compromise OMVs’ integrity and reduce immunogenicity [91,92]. Alternatively, bacterial genetic modification of lipid A synthesis to produce low-acylated lipid A may reduce naturally released OMV’s toxicity, eliminating the need for detergents [27,93]. Therefore, the key to the future clinical application of OMV-based tumor vaccines lies in balancing their immunogenicity as an adjuvant with the risk of eliciting excessive and uncontrollable immune responses.

### Low yield

OMV production often involves ultracentrifugation and ultrafiltration to prevent contamination, but these methods yield less than 100 ng/10^8^ CFU, while protein precipitation yields about ten times more, which is insufficient for developing OMVs as clinically viable delivery systems and is time-consuming as well [94,95]. Additionally, detergent treatments can help purify OMVs but may affect their natural structure [96]. To solve this issue, it is practical to enhance OMV production through genetic engineering of Gram-negative bacterial OM attachment strength such as knockout of the *Lpp* gene or creating stress-induced cultivation environments [97,98]. In addition, advanced extraction techniques such as magnetic harvesting could be developed [99]. Finally, it is essential to develop quality control standards and safety assessment methods for OMV vaccines during production to ensure safety, potency, and batch-to-batch consistency of OMV vaccines.

### Low expression level of tumor antigens

Research on OMV-based breast cancer vaccine found that only 1% of the OMV surface displays the targeted antigens [46]. Although this expression level can effectively initiate an immune response, the immune response would be more robust if increasing the efficiency of antigen delivery to APCs through higher antigen presentation level on OMVs [87]. Therefore, it is essential to enhance the antigen load and delivery efficiency of OMV-based tumor vaccines through advanced engineering methods such as genetic engineering or chemical modifications.

### Susceptibility to triggering unexpected reactions

OMVs may contain immune-dominant antigens that can misdirect immune responses, as well as molecules that are immunosuppressive or otherwise hinder protective immunity [20]. Therefore, it is crucial to design OMV-based vaccines that steer the immune system toward therapeutic responses while minimizing or avoiding adverse effects.

## Conclusion

In conclusion, OMVs represent a promising and versatile platform for the development of tumor vaccines, offering significant advantages such as strong immunogenicity, efficient antigen delivery, and engineering potential. While substantial advancements have been made in engineering OMVs to enhance tumor antigen presentation and improve their therapeutic efficacy, challenges remain, particularly in addressing issues related to reactogenicity, antigen expression, and tumor heterogeneity. Moving forward, future research should focus on optimizing OMV engineering strategies, ensuring safety, and exploring personalized vaccine formulations to improve clinical outcomes. With continued innovation and refinement, OMV-based vaccines hold great potential for advancing cancer immunotherapy and transforming the landscape of tumor vaccination.

**Summary**

- Outer membrane vesicles (OMVs) offer a transformative platform in cancer immunotherapy due to their strong immunogenicity, effective delivery capacity, and versatile engineering capabilities.
- OMV-based tumor vaccines still face challenges such as high reactogenicity of pathogen-associated molecular patterns, low yield, low expression level of tumor antigens, and susceptible to triggering unexpected reactions.
- Future research should focus on optimizing OMV engineering, improving production technologies, establishing evaluation methods and developing quality control measures for clinical application.

## Abbreviations

ABC: ATP-binding cassette
ADCC: antibody-dependent cellular cytotoxicity
APCs: antigen-presenting cells
BBB: blood-brain barrier
CDT: chemodynamic therapy
CTL: cytotoxic T lymphocyte
CXCL: C-X-C motif chemokine ligand
ClyA: cytolysin A
DAMPs: damage-associated molecular patterns
DCs: dendritic cells
DOX: doxorubicin
GFP: green fluorescent protein
HPV: human papillomavirus
Hbp: hemoglobin protease
ICD: immunogenic cell death
IFN-γ: interferon-γ
IL: interleukin
LPS: lipopolysaccharide
1-MT: 1-methyltryptophan
MTD: metronidazole
NETs: neutrophil extracellular traps
OM: outer membrane
OMVs: outer membrane vesicles
PAMPs: pathogen-associated molecular patterns
PDT: photodynamic therapy
PQS: Pseudomonas quinolone signal
PTT: photothermal therapy
TAM: tumor-associated macrophage
TLR: toll-like receptor
TME: tumor microenvironment
Tat: twin-arginine translocation
tAg: tumor antigens

## References

[1] Sung H., Ferlay J., Siegel R.L., Laversanne M., Soerjomataram I., Jemal A. (2021). Global cancer statistics 2020: globocan estimates of incidence and mortality worldwide for 36 cancers in 185 countries. CA. Cancer J. Clin..

[2] Hanahan D., Weinberg R.A (2011). Hallmarks of cancer: the next generation. Cell.

[3] Chen D.S., Mellman I (2017). Elements of cancer immunity and the cancer-immune set point. Nature.

[4] Vanneman M., Dranoff G (2012). Combining immunotherapy and targeted therapies in cancer treatment. Nat. Rev. Cancer.

[5] Melief C.J.M.,, van Hall, T.,, Arens R., Ossendorp F., van der Burg S.H. (2015). Therapeutic cancer vaccines. J. Clin. Invest..

[6] Klebanoff C.A., Acquavella N., Yu Z., Restifo N.P (2011). Therapeutic cancer vaccines: are we there yet?. Immunol. Rev..

[7] Fan T., Zhang M., Yang J., Zhu Z., Cao W., Dong C (2023). Therapeutic cancer vaccines: advancements, challenges, and prospects. Signal Transduct. Target. Ther..

[8] Luo Z.H., Cheng X., Feng B., Fan D.Y., Liu X., Xie R.Y. (2024). Engineering versatile bacteria-derived outer membrane vesicles: an adaptable platform for advancing cancer immunotherapy. Adv. Sci. (Weinh)..

[9] Knox K.W., Vesk M., Work E (1966). Relation between excreted lipopolysaccharide complexes and surface structures of a lysine-limited culture of *Escherichia coli*. J. Bacteriol..

[10] Schwechheimer C., Kuehn M.J (2015). Outer-membrane vesicles from gram-negative bacteria: biogenesis and functions. Nat. Rev. Microbiol..

[11] Kulp A., Kuehn M.J (2010). Biological functions and biogenesis of secreted bacterial outer membrane vesicles. Annu. Rev. Microbiol..

[12] Keenan J.I., Davis K.A., Beaugie C.R., McGovern J.J., Moran A.P (2008). Alterations in *Helicobacter pylori* outer membrane and outer membrane vesicle-associated lipopolysaccharides under iron-limiting growth conditions. Innate Immun..

[13] Turner L., Praszkier J., Hutton M.L., Steer D., Ramm G., Kaparakis-Liaskos M (2015). Increased Outer Membrane Vesicle Formation in a Helicobacter pylori tolB Mutant. Helicobacter.

[14] Kesty N.C., Mason K.M., Reedy M., Miller S.E., Kuehn M.J (2004). Enterotoxigenic *Escherichia coli* vesicles target toxin delivery into mammalian cells. EMBO J..

[15] Renelli M., Matias V., Lo R.Y., Beveridge T.J (2004). DNA-containing membrane vesicles of *Pseudomonas aeruginosa* PAO1 and their genetic transformation potential. Microbiology (Reading, Engl.).

[16] Kaparakis M., Turnbull L., Carneiro L., Firth S., Coleman H.A., Parkington H.C. (2010). Bacterial membrane vesicles deliver peptidoglycan to NOD1 in epithelial cells. Cell. Microbiol..

[17] Bernadac A., Gavioli M., Lazzaroni J.C., Raina S., Lloubès R (1998). Escherichia coli tol-pal Mutants Form Outer Membrane Vesicles. J. Bacteriol.

[18] Irving A.T., Mimuro H., Kufer T.A., Lo C., Wheeler R., Turner L.J. (2014). The immune receptor NOD1 and kinase RIP2 interact with bacterial peptidoglycan on early endosomes to promote autophagy and inflammatory signaling. Cell Host Microbe.

[19] Coley W.B (1991). The Classic - the treatment of malignant-tumors by repeated inoculations of erysipelas - with a report of 10 original cases. Clin Orthop Relat R.

[20] Zhang Y.X., Fang Z.Y., Li R.Z., Huang X.T., Liu Q (2019). Design of outer membrane vesicles as cancer vaccines: a new toolkit for cancer therapy. Cancers (Basel).

[21] DeVoe I.W., Gilchrist J.E (1975). Pili on meningococci from primary cultures of nasopharyngeal carriers and cerebrospinal fluid of patients with acute disease. J. Exp. Med..

[22] Liu G.N., Ma N.A., Cheng K.M., Feng Q.Q., Ma X.T., Yue Y.L. (2024). Bacteria-derived nanovesicles enhance tumour vaccination by trained immunity. Nat. Nanotechnol..

[23] Kim O.Y., Park H.T., Dinh N.T.H., Choi S.J., Lee J., Kim J.H. (2017). Bacterial outer membrane vesicles suppress tumor by interferon-γ-mediated antitumor response. Nat. Commun..

[24] Bomberger J.M., Maceachran D.P., Coutermarsh B.A., Ye S., O’Toole G.A., Stanton B.A (2009). Long-distance delivery of bacterial virulence factors by *Pseudomonas aeruginosa* outer membrane vesicles. Plos Pathog..

[25] Elhenawy W., Debelyy M.O., Feldman M.F (2014). Preferential packing of acidic glycosidases and proteases into bacteroides outer membrane vesicles. MBio.

[26] Berleman J.E., Allen S., Danielewicz M.A., Remis J.P., Gorur A., Cunha J. (2014). The lethal cargo of *Myxococcus xanthus* outer membrane vesicles. Front. Microbiol..

[27] Sartorio M.G., Pardue E.J., Feldman M.F., Haurat M.F (2021). Bacterial outer membrane vesicles: from discovery to applications. Annu. Rev. Microbiol..

[28] Haurat M.F., Aduse-Opoku J., Rangarajan M., Dorobantu L., Gray M.R., Curtis M.A. (2011). Selective sorting of cargo proteins into bacterial membrane vesicles. J. Biol. Chem..

[29] Delacour D., Greb C., Koch A., Salomonsson E., Leffler H., Le Bivic A. (2007). Apical sorting by galectin-3-dependent glycoprotein clustering. Traffic.

[30] Valguarnera E., Scott N.E., Azimzadeh P., Feldman M.F (2018). Surface exposure and packing oflLipoproteins into outer membrane vesicles are coupled processes in b*acteroides*. mSphere.

[31] Fusciello M., Fontana F., Tähtinen S., Capasso C., Feola S., Martins B. (2019). Artificially cloaked viral nanovaccine for cancer immunotherapy. Nat. Commun..

[32] Luo M., Samandi L.Z., Wang Z.H., Chen Z.J.J., Gao J.M (2017). Synthetic nanovaccines for immunotherapy. J. Control. Release.

[33] Hegde S., Krisnawan V.E., Herzog B.H., Zuo C., Breden M.A., Knolhoff B.L. (2020). Dendritic cell paucity leads to dysfunctional immune surveillance in pancreatic cancer. Cancer Cell.

[34] DeNardo D.G., Ruffell B (2019). Macrophages as regulators of tumour immunity and immunotherapy. Nat. Rev. Immunol..

[35] Ding J.Q., Sui D.Z., Liu M.Q., Su Y.Q., Wang Y., Liu M.Y. (2021). Sialic acid conjugate-modified liposomes enable tumor homing of epirubicin via neutrophil/monocyte infiltration for tumor therapy. Acta Biomater..

[36] Komohara Y., Fujiwara Y., Ohnishi K., Takeya M (2016). Tumor-associated macrophages: potential therapeutic targets for anti-cancer therapy. Adv. Drug Deliv. Rev..

[37] Tavano R., Franzoso S., Cecchini P., Cartocci E., Oriente F., Aricò B. (2009). The membrane expression of *Neisseria meningitidis* adhesin A (NadA) increases the proimmune effects of MenB OMVs on human macrophages, compared with NadA- OMVs, without further stimulating their proinflammatory activity on circulating monocytes. J. Leukoc. Biol..

[38] Kaparakis-Liaskos M., Ferrero R.L (2015). Immune modulation by bacterial outer membrane vesicles. Nat. Rev. Immunol..

[39] Wang X.G., Eagen W.J., Lee J.C (2020). Orchestration of human macrophage NLRP3 inflammasome activation by extracellular vesicles. P Natl Acad Sci U.S.A..

[40] Mi Z., Yao Q., Qi Y., Zheng J., Liu J., Liu Z. (2023). Salmonella-mediated blood‒brain barrier penetration, tumor homing and tumor microenvironment regulation for enhanced chemo/bacterial glioma therapy. Acta Pharm. Sin. B.

[41] Tanaka T., Narazaki M., Kishimoto T (2018). Interleukin (IL-6) Immunotherapy. Cold Spring Harb. Perspect. Biol..

[42] Grouard G., Durand I., Filgueira L., Banchereau J., Liu Y.J (1996). Dendritic cells capable of stimulating T cells in germinal centres. Nature.

[43] Wang S.H., Gao J., Li M., Wang L.G., Wang Z.J (2018). A facile approach for development of a vaccine made of bacterial double-layered membrane vesicles (DMVs). Biomaterials.

[44] Grotzke J.E., Kozik P., Morel J.D., Impens F., Pietrosemoli N., Cresswell P. (2017). Sec61 blockade by mycolactone inhibits antigen cross-presentation independently of endosome-to-cytosol export. Proc. Natl. Acad. Sci. U.S.A..

[45] Tang-Huau T.L., Gueguen P., Goudot C., Durand M., Bohec M., Baulande S. (2018). Human in vivo-generated monocyte-derived dendritic cells and macrophages cross-present antigens through a vacuolar pathway. Nat. Commun..

[46] Wang S.J., Huang W.W., Li K., Yao Y.F., Yang X., Bai H.M (2017). Engineered outer membrane vesicle is potent to elicit HPV16E7-specific cellular immunity in a mouse model of TC-1 graft tumor. Int. J. Nanomedicine.

[47] Vaughan A.T., Brackenbury L.S., Massari P., Davenport V., Gorringe A., Heyderman R.S. (2010). Selectively induces mitogenic proliferation of the naive B cell pool via cell surface Ig. J. Immunol..

[48] Andersen M.H., Schrama D., Thor Straten P., Becker J.C (2006). Cytotoxic T cells. J. Invest. Dermatol..

[49] Gao J., Su Y.J., Wang Z.J (2022). Engineering bacterial membrane nanovesicles for improved therapies in infectious diseases and cancer. Adv. Drug Deliv. Rev..

[50] Bachmann M.F., Jennings G.T (2010). Vaccine delivery: a matter of size, geometry, kinetics and molecular patterns. Nat. Rev. Immunol..

[51] Gujrati V., Kim S., Kim S.H., Min J.J., Choy H.E., Kim S.C. (2014). Bioengineered bacterial outer membrane vesicles as cell-specific drug-delivery vehicles for cancer therapy. ACS Nano.

[52] Peng L.H., Wang M.Z., Chu Y., Zhang L., Niu J., Shao H.T. (2020). Engineering bacterial outer membrane vesicles as transdermal nanoplatforms for photo-TRAIL-programmed therapy against melanoma. Sci. Adv..

[53] Yue Y.L., Xu J.Q., Li Y., Cheng K.M., Feng Q.Q., Ma X.T. (2022). Antigen-bearing outer membrane vesicles as tumour vaccines produced *in situ* by ingested genetically engineered bacteria. Nat Biomed Eng.

[54] Shen X.Y., Zhu C.J., Liu X.T., Zheng H.Q., Wu Q., Xie J.J. (2023). Engineered bacteria for augmented tumor vaccination. Biomater Sci-Uk.

[55] Gerritzen M.J.H., Martens D.E., Wijffels R.H., Stork M (2017). Bioengineering bacterial outer membrane vesicles as vaccine platform. Biotechnol. Adv..

[56] Galen J.E., Curtiss R (2014). The delicate balance in genetically engineering live vaccines. Vaccine (Auckl)..

[57] Leo J.C., Grin I., Linke D (2012). Type V secretion: mechanism(s) of autotransport through the bacterial outer membrane. Phil. Trans. R. Soc. B.

[58] Jong W.S.P., Slotboom D.J., Tame J.R.H., Wickström D. (2007). Limited tolerance towards folded elements during secretion of the autotransporter Hbp. Mol. Microbiol..

[59] Luirink J., Jong W.S.P (2016). Fusion protein for secretory protein expression.

[60] Kim J.Y., Doody A.M., Chen D.J., Cremona G.H., Shuler M.L., Putnam D. (2008). Engineered bacterial outer membrane vesicles with enhanced functionality. J. Mol. Biol..

[61] Wai S.N., Lindmark B., Söderblom T., Takade A., Westermark M., Oscarsson J. (2003). Vesicle-mediated export and assembly of pore-forming oligomers of the enterobacterial ClyA cytotoxin. Cell.

[62] Chen D.J., Osterrieder N., Metzger S.M., Buckles E., Doody A.M., DeLisa M.P. (2010). Delivery of foreign antigens by engineered outer membrane vesicle vaccines. Proc. Natl. Acad. Sci. U.S.A.

[63] Kesty N.C., Kuehn M.J (2004). Incorporation of heterologous outer membrane and periplasmic proteins into *Escherichia coli* outer membrane vesicles. J. Biol. Chem..

[64] Pautsch A., Schulz G.E (1998). Structure of the outer membrane protein A transmembrane domain. Nat. Struct. Biol..

[65] Kim S.H., Kim K.S., Lee S.R., Kim E., Kim M.S., Lee E.Y. (2009). Structural modifications of outer membrane vesicles to refine them as vaccine delivery vehicles. Biochim. Biophys. Acta.

[66] Cheng K.M., Zhao R.F., Li Y., Qi Y.Q., Wang Y.Z., Zhang Y.L. (2021). Bioengineered bacteria-derived outer membrane vesicles as a versatile antigen display platform for tumor vaccination via plug-and-display technology. Nat. Commun..

[67] Zakeri B., Fierer J.O., Celik E., Chittock E.C., Schwarz-Linek U., Moy V.T. (2012). Peptide tag forming a rapid covalent bond to a protein, through engineering a bacterial adhesin. Proc. Natl. Acad. Sci. U.S.A..

[68] Veggiani G., Nakamura T., Brenner M.D., Gayet R.V., Yan J., Robinson C.V. (2016). Programmable polyproteams built using twin peptide superglues. Proc. Natl. Acad. Sci. U.S.A..

[69] Li Y., Ma X.T., Yue Y.L., Zhang K.Y., Cheng K.M., Feng Q.Q. (2022). Rapid surface display of mRNA antigens by bacteria‐derived outer membrane vesicles for a personalized tumor vaccine. Adv. Mater. Weinheim.

[70] Huang L., Ashraf S., Lilley D.M.J (2019). The role of RNA structure in translational regulation by L7Ae protein in archaea. RNA.

[71] Hamon M.A., Ribet D., Stavru F., Cossart P (2012). Listeriolysin O: the Swiss army knife of *Listeria*. Trends Microbiol..

[72] Li L., Zou J., Dai Y.L., Fan W.P., Niu G., Yang Z. (2020). Burst release of encapsulated annexin A5 in tumours boosts cytotoxic T-cell responses by blocking the phagocytosis of apoptotic cells. Nat. Biomed. Eng..

[73] Li Y.J., Wu J.Y., Qiu X.H., Dong S.H., He J., Liu J.H. (2023). Bacterial outer membrane vesicles-based therapeutic platform eradicates triple-negative breast tumor by combinational photodynamic/chemo-/immunotherapy. Bioact. Mater..

[74] Liu X.M., Sun M.Y., Pu F., Ren J.S., Qu X.G (2023). Transforming intratumor bacteria into immunopotentiators to reverse cold tumors for enhanced immuno-chemodynamic therapy of triple-negative breast cancer. J. Am. Chem. Soc..

[75] Min Y.Z., Roche K.C., Tian S.M., Eblan M.J., McKinnon K.P., Caster J.M. (2021). Author correction: antigen-capturing nanoparticles improve the abscopal effect and cancer immunotherapy. Nat. Nanotechnol..

[76] Li Y., Zhang K.Y., Wu Y., Yue Y.L., Cheng K.M., Feng Q.Q. (2022). Antigen capture and immune modulation by bacterial outer membrane vesicles as *in situ* vaccine for cancer immunotherapy post‐photothermal therapy. Small.

[77] Yu B., Goel S., Ni D., Ellison P.A., Siamof C.M., Jiang D. (2018). Reassembly of Zr-labeled cancer cell membranes into multicompartment membrane-derived liposomes for PET-trackable tumor-targeted theranostics. Adv. Mater. Weinheim.

[78] Fang L., Zhao Z.T., Wang J., Zhang P.C., Ding Y.P., Jiang Y.Y. (2020). Engineering autologous tumor cell vaccine to locally mobilize antitumor immunity in tumor surgical bed. Sci. Adv..

[79] Chen Q., Huang G.J., Wu W.T., Wang J.W., Hu J.W., Mao J.M. (2020). A hybrid eukaryotic–prokaryotic nanoplatform with photothermal modality for enhanced antitumor vaccination. Adv. Mater. Weinheim.

[80] Zou M.Z., Li Z.H., Bai X.F., Liu C.J., Zhang X.Z (2021). Hybrid vesicles based on autologous tumor cell membrane and bacterial outer membrane to enhance innate immune response and personalized tumor immunotherapy. Nano Lett..

[81] Vesikari T., Esposito S., Prymula R., Ypma E., Kohl I., Toneatto D. (2013). Immunogenicity and safety of an investigational multicomponent, recombinant, meningococcal serogroup B vaccine (4CMenB) administered concomitantly with routine infant and child vaccinations: results of two randomised trials. Lancet.

[82] Johnson B (2023). GSK’s gonorrhea vaccine receives fast-track designation to expedite clinical trials. Nat. Med..

[83] Camacho A.I.,, de Souza, J.,, Sánchez-Gómez S., Pardo-Ros M., Irache J.M., Gamazo C (2011). Mucosal immunization with Shigella flexneri outer membrane vesicles induced protection in mice. Vaccine (Auckl)..

[84] Roier S., Leitner D.R., Iwashkiw J., Schild-Prüfert K., Feldman M.F., Krohne G. (2012). Intranasal immunization with nontypeable Haemophilus influenzae outer membrane vesicles induces cross-protective immunity in mice. Plos one.

[85] Drabick J.J., Brandt B.L., Moran E.E., Saunders N.B., Shoemaker D.R., Zollinger W.D (1999). Safety and immunogenicity testing of an intranasal group B meningococcal native outer membrane vesicle vaccine in healthy volunteers. Vaccine (Auckl)..

[86] Haneberg B., Dalseg R., Wedege E., Høiby E.A., Haugen I.L., Oftung F. (1998). Intranasal administration of a meningococcal outer membrane vesicle vaccine induces persistent local mucosal antibodies and serum antibodies with strong bactericidal activity in humans. Infect. Immun..

[87] Wang S.M., Guo J.Y., Bai Y., Sun C., Wu Y.H., Liu Z. (2022). Bacterial outer membrane vesicles as a candidate tumor vaccine platform. Front. Immunol..

[88] Rappuoli R., Pizza M., Masignani V., Vadivelu K (2018). Meningococcal B vaccine (4CMenB): the journey from research to real world experience. Expert Rev. Vaccines.

[89] van der Pol L.,, Stork M, van der Ley P (2015). Outer membrane vesicles as platform vaccine technology. Biotechnol. J..

[90] Gnopo Y.M.D., Watkins H.C., Stevenson T.C., DeLisa M.P., Putnam D (2017). Designer outer membrane vesicles as immunomodulatory systems - reprogramming bacteria for vaccine delivery. Adv. Drug Deliv. Rev..

[91] van de Waterbeemd B, Mommen G.P.M., Pennings J.L.A., Eppink M.H., Wijffels R.H.,, van der Pol L.A. (2013). Quantitative proteomics reveals distinct differences in the protein content of outer membrane vesicle vaccines. J. Proteome Res..

[92] Ferrari G., Garaguso I., Adu-Bobie J., Doro F., Taddei A.R., Biolchi A. (2006). Outer membrane vesicles from group B *Neisseria meningitidis* delta gna33 mutant: proteomic and immunological comparison with detergent-derived outer membrane vesicles. Proteomics.

[93] Choi D.S., Kim D.K., Choi S.J., Lee J., Choi J.P., Rho S. (2011). Proteomic analysis of outer membrane vesicles derived from *Pseudomonas aeruginosa*. Proteomics.

[94] Li M., Zhou H., Yang C., Wu Y., Zhou X., Liu H. (2020). Bacterial outer membrane vesicles as a platform for biomedical applications: an update. J. Control. Release.

[95] Jain S., Pillai J (2017). Bacterial membrane vesicles as novel nanosystems for drug delivery. Int. J. Nanomedicine.

[96] van de Waterbeemd, B.,, Streefland M.,, van der Ley, P.,, Zomer B.,, van Dijken, H.,, Martens D. (2010). Improved OMV vaccine against Neisseria meningitidis using genetically engineered strains and a detergent-free purification process. Vaccine (Auckl)..

[97] McBroom A.J., Johnson A.P., Vemulapalli S., Kuehn M.J (2006). Outer membrane vesicle production by *Escherichia coli* is independent of membrane instability. J. Bacteriol..

[98] Klimentová J., Stulík J (2015). Methods of isolation and purification of outer membrane vesicles from gram-negative bacteria. Microbiol. Res..

[99] Shi R., Dong Z., Ma C., Wu R., Lv R., Liu S. (2022). High-yield, magnetic harvesting of extracellular outer-membrane vesicles from *Escherichia coli*. Small.

